# The Enhancing Role of Emotion Regulation in the Links between Early Positive Memories and Self-harm and Suicidal Ideation in Adolescence

**DOI:** 10.1007/s10964-023-01777-8

**Published:** 2023-05-13

**Authors:** Célia Barreto Carvalho, Marco Teixeira, Rodrigo Costa, Filipa Cordeiro, Joana Moura Cabral

**Affiliations:** 1 Faculty of Social and Human Sciences, University of the Azores, Ponta Delgada, Portugal; 2 Cognitive and Behavioural Centre for Research and Intervention, Faculty of Psychology and Education Sciences, University of Coimbra, Coimbra, Portugal; 3 Family Therapy and Systemic Intervention Center, Coimbra, Portugal; 4 Gaspar Frutuoso Foundation, University of the Azores, Ponta Delgada, Portugal

**Keywords:** Adolescence, Early memories of warmth and safeness, Emotion regulation, Self-harm, Suicidal ideation

## Abstract

Adolescence is a critical developmental period associated with an increased variety of interrelated risks and vulnerabilities. Previous studies have found associations between early memories of warmth and safeness, as well as emotion regulation, and self-harm and suicidal ideation in adolescence. Additionally, these early emotional memories have been found to be positively linked with some indicators of emotion regulation during this period. The present cross-sectional study extends prior research by exploring the moderating role of emotion regulation in the relationships between early memories of warmth and safeness, as well as each of the following risk-related outcomes in adolescence, in younger (i.e., 13–15) and older (i.e., 16–19) adolescents: suicidal ideation and self-harm and its associated functions (i.e., automatic and social reinforcement. Three self-report measures of these early emotional memories, emotion regulation, and risk-related outcomes, and a sample of 7918 Portuguese adolescents (53.3% females), with ages ranging from 13 to 19 (*M*_age_ = 15.5), were used. In both age groups, at high levels of emotion regulation, early memories of warmth and safeness had a greater (negative) effect on suicidal ideation and the automatic reinforcement function of self-harm, compared to at average and low levels of emotion regulation. These findings highlight the enhancing role of emotion regulation on the associations between early memories of warmth and safeness and some risk-related outcomes in adolescents, both younger and older, which reveals the relevance of targeting emotion regulation when preventing or tackling these outcomes, regardless of adolescents’ levels of early memories of warmth and safeness.

## Introduction

Previous studies have established negative associations between early positive emotional memories and self-harm and suicidal ideation in adolescence (Barreto Carvalho et al., 2015, 2017). Emotion regulation has also been associated with each of these risk-related outcomes during this developmental period (e.g., Brausch et al., 2022); however, most of these studies have focused on the negative effect of emotion dysregulation, with limited research assessing the protective effect of adaptive emotion regulation. Furthermore, early memories of warmth and safeness have been found to be positively linked with some indicators of emotion regulation in adolescence (e.g., Marta-Simões & Ferreira, 2020). Taken together, these findings indicate a possible interaction between early memories of warmth and safeness and emotion regulation playing a protective role against adolescent self-harm and suicidal ideation, with this interaction effect possibly varying (e.g., in intensity) in relation to the specific developmental period of adolescence (e.g., early, late). Therefore, this study examines the moderating role of emotion regulation in the associations between these early emotional memories and self-harm and suicidal ideation, both in younger (i.e., 13–15) and older adolescents (i.e., 16–19).

### Adolescence, Self-harm, and Suicidal Behavior

Adolescence is a critical biopsychosocial developmental period (Dahl et al., 2018) ranging from 10 to 19 years old (World Health Organization [WHO], n.d.) during which identity formation is pivotal and constitutes a developmental task according to some developmental theories (Erikson, 1950, 1968; Marcia, 1966)—even though this process occurs across the whole lifespan. More specifically, Erikson’s (1950, 1968) theory of psychosocial development posits that adolescents face a task entitled identity vs. identity confusion during which they must explore who they would like to become in society; they may resolve this task successfully, which results in an established and meaningful sense of self and direction, or not. Extending on this theory, Marcia’s (1966) identity status paradigm posits that in addition to these poles, two dimensions of identity formation should be considered—firstly, exploration of multiple identity alternatives and a subsequent commitment to one—according to which adolescents may fall under one of four identity statuses: identity diffusion, foreclosure, moratorium, and identity achievement. In addition to these models, developmental research has also focused on the neurocognitive aspects of adolescent development; for example, the Dual Systems Models have been proposed and posit that the brain structures mostly related to regulatory competence (i.e., prefrontal cortex) undergo maturation from early to late adolescence—reaching full maturity in early adulthood—whereas those related to emotional arousability and reward orientation (i.e., limbic system) are fully developed starting from the earlier stages of adolescence (Steinberg, 2005, 2008). These developmental characteristics are linked to the fact that adolescence is associated with an increased variety of risks and vulnerabilities (Zanus et al., 2021), including deliberate self-harm (e.g., Xiao et al., 2022) and suicidal behavior (e.g., Bilsen, 2018).

Deliberate self-harm, or non-suicidal self-injury, usually found to be higher in females (for a meta-analysis, see Bresin & Schoenleber, 2015) and declining across adolescence (Monto et al., 2018), is defined as the deliberate, direct, and socially unacceptable destruction of body tissue (e.g., skin cutting, skin burning, hitting oneself) without a suicidal intent (Esposito et al., 2019). One of the most common explanatory models of self-harm is the functional approach (Nock & Prinstein, 2004), which posits that there are four different functions grouped into automatic reinforcement and social reinforcement. The former (i.e., automatic reinforcement function)—which has been described as the most frequent function of self-harm in youth (e.g., Doyle et al., 2017)—serves the purposes of alleviating negative emotional states (e.g., “to stop feeling bad”) or creating a desirable physiological state (e.g., “to feel something”). The social reinforcement function translates into using self-harm to modify or regulate the social environment, by either escaping interpersonal demands (e.g., avoiding punishment) or gaining something from others (e.g., receiving attention) (Nock & Prinstein, 2004). Previous research using adolescent samples shows no gender differences regarding self-harm functions (e.g., Calvete et al., 2015). In Portugal, where the present research was conducted, rates of adolescent self-harm were found to have increased from 19.6% in 2018 to 24.6% in 2022 (Equipa Aventura Social, 2022), with another study (Barreto Carvalho et al., 2017) having found a rate of 29.5%. Suicidal ideation in adolescence—a major precursor of further suicidal behavior (Miranda et al., 2014) and usually found to be higher in females and increasing across adolescence (e.g., Voss et al., 2019)—is relatively high, with one study using a representative sample of adolescents across 82 countries (Biswas et al., 2020) having found a rate of 14% and a Portuguese study (Barreto Carvalho et al., 2015) a rate of 22%.

### Early Positive Experiences and Memories, and Relations to Self-harm and Suicidal Ideation

Childhood experiences, particularly those related to feelings of threat or safeness, play an important role in subsequent emotional and social development (Gilbert & Perris, 2000), including outcomes such as self-harm and suicidal ideation. More specifically, less paternal emotional warmth has been associated with self-harm (Ran et al., 2021) and other positive parenting qualities (e.g., maternal warmth, involvement) with a decreased risk of adolescent suicidal ideation and suicidal behavior (e.g., Gorostiaga et al., 2019). When examining the effects of early experiences on developmental outcomes, it has been suggested that it is more important to specifically consider the recall of how one felt in relation to the behavior of others than just the recall of others’ behavior (Gilbert et al., 2003). Early memories of warmth and safeness correspond to the recall of an individual’s own inner positive feelings, emotions, and experiences in childhood (Richter et al., 2009), with one study (Vagos et al., 2017) having found no gender differences in adolescence and another having found that females show higher levels of these memories (Tahirović & Jusić, 2016). These memories have been found to be higher in younger (12–13) compared to older adolescents (14–15, 16–18) (Xavier et al., 2016). In addition, some Portuguese studies using adolescent samples (Barreto Carvalho et al., 2015, 2017) found negative relationships between these early emotional memories and self-harm, both its functions, and suicidal ideation.

### Emotion Regulation and Relations to Self-harm and Suicidal Ideation

Emotion regulation is a set of processes used to evaluate, modulate, and change emotional responses (Gratz & Roemer, 2004; McRae & Gross, 2020), with no gender differences having been found regarding its adaptive form (Duarte et al., 2015). Although essential across all developmental periods (Helion et al., 2019), this self-regulatory skill becomes critical in adolescence—characterized by multiple changes leading to higher emotional instability and intensity (Silk et al., 2003)—with previous research showing that the age ranges between 12/13 and 15 exhibit lower adaptive emotion regulation compared to younger (i.e., 10–11) and older adolescents (i.e., 16–19) (Cracco et al., 2017). Furthermore, limited access to adaptive emotion regulation strategies has been associated with adolescent suicidal ideation (e.g., Brausch et al., 2022; Quintana-Orts et al., 2020) and self-harm (e.g., Brausch et al., 2022; Peh et al., 2017), which suggests that adolescents’ low levels of this trait are associated with the use of self-harm to regulate emotions (Ford & Gómez, 2015).

### Early Positive Experiences and Memories and Emotion Regulation

Positive parental behavior leads to experiences of safeness in childhood, which have been found to have a positive effect on regulating affective states (Gilbert et al., 2006). Indeed, the recall of parental warmth is positively linked with the ability to be self-reassuring and self-soothing in stressful situations (Irons et al., 2006), an adaptive emotion regulation process (Marta-Simões et al., 2017). Lastly, previous studies have linked early memories of warmth and safeness with multiple indicators of adaptive emotion regulation (i.e., self-compassion and feelings of social safeness and pleasure: Marta-Simões & Ferreira, 2020; secure attachment: Tahirović & Jusić, 2016).

## Current Study

The literature shows that early positive emotional memories and emotion regulation have a protective effect against adolescent self-harm and suicidal ideation; however, little is known about the possible interaction effect that the former variables have on the latter across different stages of adolescence. To address this gap, this study explored the relevance of early memories of warmth and safeness, in interaction with emotion regulation, to prevent or reduce the risk of self-harm, its functions, and suicidal ideation in younger and older adolescents. The hypotheses were based on the literature outlined previously. The first aim of the present study was to characterize the study variables (i.e., early memories of warmth and safeness, emotion regulation, self-harm, its automatic and social reinforcement functions, suicidal ideation), including the prevalence of self-harm and suicidal ideation, in the total sample and by gender and specific age group (i.e., 13, 14, 15, 16, 17, 18 or older). It was hypothesized that self-harm and suicidal ideation would be higher in females, and that there would be no differences regarding both functions of self-harm and emotion regulation (Hypothesis 1a). It was also hypothesized that early memories of warmth and safeness and self-harm would be higher in younger age groups and that suicidal ideation and emotion regulation higher in older age groups (Hypothesis 1b). The second aim was to examine the associations between gender, age, early memories of warmth and safeness, emotion regulation, self-harm and its functions, and suicidal ideation. In line with the above, it was hypothesized that gender would be positively associated with self-harm and suicidal ideation and that it would not be associated with any functions of self-harm nor emotion regulation (Hypothesis 2a). Furthermore, it was hypothesized that age would be negatively associated with early memories of warmth and safeness and self-harm, as well as positively associated with suicidal ideation and emotion regulation (Hypothesis 2b). It was also hypothesized that positive associations would be found between emotion regulation and early memories of warmth and safeness, and negative associations between each of these and adolescent self-harm, its functions, and suicidal ideation (Hypothesis 2c). Lasty, as said earlier, the primary aim of this study was to explore the moderation of emotion regulation on the relationships between early memories of warmth and safeness and self-harm, its functions, and suicidal ideation in adolescence, in younger (i.e., 13–15) and older (i.e., 16–19) age groups. It was hypothesized that in both age groups, higher levels of emotion regulation would have a positive effect on the (negative) associations between early memories of warmth and safeness and each of the risk-related outcomes (Hypothesis 3).

## Methods

### Participants

The sample of the present study is part of a research project entitled Vida + – described below (in the “Procedure and Ethics” section)—and is composed of nearly the totality of students enrolled in the Portuguese public education system (*ensino regular público*) living on all the islands (i.e., nine) of the Autonomous Region of the Azores, Portugal. The initial sample comprised a total of 8622 individuals, of which 704 were excluded because they either did not report their age, were below the age of 13 or older than 19—considering the small number of adolescents in both groups (*n* = 249) and in line with the definition of adolescence according to WHO (n.d.)—and/or did not report their school year or were in fourth or fifth grade—given the small size of this subsample (*n* = 2). This led to a final sample of 7918 adolescents, of which 3697 (46.7%) are male and 4218 (53.3%) are female, with ages ranging from 13 to 19 (*M* = 15.5, *SD* = 1.7). At time of participation, most participants were in ninth grade (24.9%), seventh grade (22.1%), or eighth grade (21.1%), and had never failed a school year (64.6%). No information was collected regarding participants’ racial/ethnic characteristics nor socioeconomic status. The sociodemographic characteristics of the study are presented in Table 1.

**Table 1:** Participants’ Sociodemographic Characteristics (*N* = 7918)

Sociodemographic characteristics	Sample	
	*n*	*%*
Gender		
Male	3697	46.7
Female	4218	53.3
Age groups		
13 years	1185	15
14 years	1403	17.7
15 years	1713	21.6
16 years	1385	17.5
17 years	1103	13.9
≥18 years	1129	14.3
School year^a^		
6th grade (11 years old)	48	0.6
7th grade (12 years old)	1752	22.1
8th grade (13 years old)	1673	21.1
9th grade (14 years old)	1975	24.9
10th grade (15 years old)	1122	14.2
11th grade (16 years old)	780	9.9
12th grade (17 years old)	568	7.2
Ever failed a school year		
Yes	2593	35.4
No	5734	64.6

^a^The ages most commonly associated with each Portuguese school year are presented in parentheses

### Measures

All data were collected using a research protocol created specifically for Vida+, the greater research project this study is part of—see the “Procedure and Ethics” section for further information on this project. The protocol included multiple self-report measures and questionnaires, three of which were used for this study and are described below.

#### Early memories of warmth and safeness

The Portuguese version of the Early Memories of Warmth and Safeness for Adolescents Scale (EMWSS-A; original version by Richter et al., 2009; Portuguese adolescent version by Cunha et al., 2014) was used to measure recall of positive feelings and experiences of safeness, contentment, and warmth in childhood. It is a unidimensional 21-item scale, measured on a 5-point Likert scale ranging from 0 = *No, never* to 4 = *Yes, most of the time*, that examines personal emotional memories—and not recall of parental behavior – related to being cared about in childhood (e.g., “I felt secure and safe”, “I felt comfortable sharing my feeling and thoughts with those around me”). Total scores for each participant were computed based on the sum of all items, with higher scores indicating higher levels of positive memories. The scale showed excellent values of internal consistency, *α* = 0.97, in the original study (Richter et al., 2009). In the Portuguese adolescent version (Cunha et al., 2014) the scale displayed an excellent internal consistency, *α* = 0.95. In this study, the scale also had an excellent internal consistency, *α* = 0.98.

#### Self-harm and suicidal ideation

The Impulse, Self-harm and Suicide Ideation Questionnaire for Adolescents (ISSIQ-A; Barreto Carvalho et al., 2015) was used to measure self-harm, suicidal ideation, as well as two functions of self-harm used by adolescents: the automatic reinforcement function (i.e., to create or alleviate emotional states) and the social function (i.e., to influence social relationships). The questionnaire comprises 56 items and is grouped into six subscales, of which only four were used given the variables of interest for the present study: self-harm (eight items; e.g., “I hurt myself or inflict pain on myself voluntarily, in other words, on purpose”, “I scratch or pinch some parts of my body on purpose”), the automatic reinforcement function of self-harm (24 items; e.g., “I hurt myself to alleviate the negative emotions I feel”, “I hurt myself to be able to feel something”), the social reinforcement function of self-harm (seven items; e.g., “Hurting myself helps other understand my problems”, “I hurt myself to get others’ attention”), and suicidal ideation (three items; e.g., “There have been times during which I thought I wanted to die”, “There are times during which I would like to disappear”). Each item is scored on a 4-point Likert scale ranging from 0 = *Never happens to me* to 3 = *Always happens to me*. A total score for each subscale was computed for all participants based on the sums of the corresponding items, with higher scores indicating greater presence of each construct/behavior. In the original study (Carvalho et al., 2015), the subscales showed acceptable to excellent values of internal consistency, ranging from *α* = 0.77 for the social reinforcement function of self-harm to *α* = 0.93 to the automatic reinforcement function of self-harm. In the present study, good to excellent values of internal consistency for the different subscales were found, ranging from *α* = 0.86 for suicidal ideation to *α* = 0.98 for the automatic reinforcement function of self-harm.

#### Emotion regulation

The Portuguese version of the Situational Test of Emotional Management—Brief (STEM-B; Allen et al., 2015; Portuguese version by da Motta et al., 2021) was used to measure emotion regulation in the form of emotional management ability. It is an 18-item short version of the original 44-item Situational Test of Emotional Management (STEM; MacCann & Roberts, 2008). Each item describes a hypothetical emotional situation (e.g., “Joana and Marina shared an office for years, but Joana got a new job and Marina lost touch with her”) in which participants are asked to choose from four responses (e.g., “Simply accept that Joana is gone and the friendship is over”, “Call Joana and invite her to lunch or drink a coffee to catch up”, “Contact Joana and invite her for a chat, but also make friends with the person who replaced her at the office”, “Get to know other people at the office and make new friendships”) the most effective course of action to manage both the emotions the person is feeling and the problems they face in each specific situation. Dichotomous scoring is used, based on the proportion of experts who selected the most appropriate answer for each item in the original validation study, with this answer scored as 1 and the remaining options as 0. A total score was computed for each participant based on the sum of all items, with higher scores indicating higher levels of emotion regulation. In the original study (Allen et al., 2015), this measure showed a good internal consistency, *α* = 0.84. In the Portuguese version (da Motta et al., 2021), an acceptable internal consistency was found, α (Kuder-Richardson Formula 20 [KR-20]) = 0.62, which was considered reasonable given that KR-20 may be influenced by the measure’s (brief) length, difficulty, and average item correlations (Cortina, 1993). In the present study, the scale also presented an acceptable internal consistency, α (KR-20) = 0.60.

### Procedure and Ethics

This study is part of a greater research project called Vida+, developed by the University of the Azores and aimed to explore individual (e.g., disruptive emotional experiences, coping strategies, emotion regulation) and specific sociocultural factors influencing substance use in the Autonomous Region of the Azores. Prior to data collection, this research project was approved by the Ethics Committee of the University of the Azores and the Portuguese Data Protection Authority (no. 13953/2017). A research protocol was developed using multiple self-report measures and questionnaires for Vida+, three of which were used for this study. In order to maximize student participation, this protocol was administered both digitally (e.g., via an electronic link) and using a paper and pencil format to students across Azorean schools on all islands of this Portuguese Autonomous Region. Participation of students was split into two different moments, as a way to prevent possible effects of fatigue on participants and maximize response accuracy.

All international ethical norms and standards regarding research involving human participants (e.g., 1964 Declaration of Helsinki and its later amendments), were complied with throughout the study, namely anonymity of data and confidentiality. Participation was voluntary and an informed consent form was signed by all participants above the age of 18 and by the underage participants’ parents or legal guardians. Collected data were stored online in the form of a dataset, in compliance with European Union’s General Data Protection Regulation (GDPR) guidelines.

#### Analytical strategy

Data were analyzed using SPSS Statistics, version 27. Descriptive statistics (e.g., means, standard deviations) were computed for all variables, both in the total sample and by gender (Hypothesis 1a) and specific age group (i.e., 13, 14, 15, 16, 17, 18 or older) (Hypothesis 1b), as well as inferential statistics (independent samples *t*-tests, ANOVAs, chi-square tests, Pearson correlations, and regression analyses). Pearson correlation analyses were conducted to examine the relationships between gender, age, early memories of warmth and safeness, emotion regulation, and the dimensions of the ISSIQ-A (i.e., self-harm, social and automatic reinforcement functions of self-harm, suicidal ideation) (Hypothesis 2a, Hypothesis 2b, and Hypothesis 2c). Lastly, linear regression analyses, specifically moderation models, were conducted in younger (i.e., 13–15) and older (i.e., 16-19) age groups, controlling for gender, using early memories of warmth and safeness as the independent variable, the dimensions of the ISSIQ-A as the dependent variables, and emotion regulation as the moderating variable, to explore if the latter (i.e., emotion regulation) significantly moderates the association between the former (i.e., early memories of warmth and safeness) and the ISSIQ-A subscales (Hypothesis 3). Gender was coded as 0 = male and 1 = female. Adolescents who answered between 1 = *Sometimes happens to me* and 3 = *Always happens to me* at least once in any item measuring self-harm were considered to display this behavior; likewise, adolescents who answered similarly in the first item measuring suicidal ideation—which directly measures this construct—were considered to show this ideation, with scores of 1 (see above), 2 = *Often happens to me*, and 3 being translated into moderate, severe, and very severe suicidal ideation, respectively. Correlation coefficients lower than 0.20 were considered weak, those between 0.20 and 0.50 were considered moderate, and those greater than 0.50 were considered strong (Ferguson, 2009). The level of significance used for all analyses was *p* < 0.05.

### Missing Data

Considering the high percentage of missing data for both the automatic and social reinforcement functions of self-harm (58.3%), two independent samples *t*-tests were conducted to compare the groups of adolescents with missing values to those with complete cases to explore whether the pattern of missingness was attributable to the presence/absence of self-harm. Indeed, those with complete cases reported higher self-harm than those with missing values, *t*(3594.44) = −30.87, *p* < 0.001. These data can be assumed to be Missing at Random (MAR) given that the missingness is related to a measured variable (Little & Rubin, 2002). The percentages of missing cases ranged from 8 to 18% for all remaining variables. Given the large sample size, missing data were excluded pairwise (i.e., correlations, *t*-tests, ANOVAs, chi-square tests) or listwise (i.e., moderations) in all analyses.

## Results

### Descriptives and Correlations

The means and standard deviations for each variable studied, including the prevalence of self-harm and suicidal ideation and the statistics for mean comparisons, are presented in Table 2. For early memories of warmth and safeness, the total sample showed a mean of 54.8; for self-harm, a mean of 1.5; for suicidal ideation, a mean of 2.1; for automatic reinforcement function of self-harm, a mean of 10.8; for social reinforcement function of self-harm, a mean of 2.6; lastly, for emotion regulation, a mean of 8.7. Over one-third of adolescents (34.5%) reported suicidal ideation, with over one-fifth (22.1%), 7.7%, and 4.7% reporting moderate, severe, and very severe suicidal ideation, respectively. Lastly, nearly one-third (28%) reported having had self-harmed at least once, with the automatic reinforcement function of self-harm (weighted *M* = 0.45) displaying relatively higher levels than the social reinforcement function (weighted *M* = 0.37).

**Table 2:** Descriptives of Early Memories of Warmth and Safeness, Self-harm and its Functions, Suicidal Ideation, and Emotion Regulation by Gender and Age Group

	EMWS-A	ISSIQ-A Self-harm		ISSIQ-A Suicidal		ISSIQ-A Automatic	ISSIQ-A Social	STEM-B
	*M* (*SD*)	*M* (*SD*)	*n* (%)	*M* (*SD*)	*n* (%)	*M* (*SD*)	*M* (*SD*)	*M* (*SD*)
Total sample	54.8 (20.5)	1.5 (3.5)	1815 (28)	2.1 (2.4)	2462 (34.5)	10.8 (14.7)	2.6 (4.4)	8.7 (3)
Gender	*t* = 2.22^*^		χ^2^ = 1.27		χ^2^ = 44.55^***^	*t* = 4.75^***^	*t* = 6.85^***^	*t* = −17.29^***^
Male	55.4 (21.1)	1.7 (3.9)	790 (27.3)	1.8 (2.3)	996 (30.4)	12.1 (16.2)	3.2 (5)	8.1 (3.1)
Female	54.3 (20)	1.3 (3.1)	1024 (28.6)	2.4 (2.4)	1465 (38)	9.7 (13.2)	2.1 (3.8)	9.3 (2.8)
Age group	*F* = 4.82^***^		χ^2^ = 24.76^***^		χ^2^ = 15.07^*^	*F* = 6.05^***^	*F* = 4.77^***^	*F* = 3.38^**^
13 years	57.4 (20.5)	1.4 (3.3)	248 (26.7)	2.9 (2.4)	312 (29.7)	11.5 (15)	3 (4.5)	8.7 (2.8)
14 years	54.5 (20.6)	1.7 (3.5)	364 (32.5)	2.1 (2.5)	429 (34.1)	12 (15)	2.8 (4.5)	8.5 (3)
15 years	54.7 (20.1)	1.7 (3.7)	400 (28.4)	2.2 (2.4)	555 (35.9)	12 (15.8)	3 (4.7)	8.8 (3)
16 years	53.4 (21.1)	1.6 (3.7)	339 (29.4)	2.3 (2.5)	460 (36.5)	10.8 (14.8)	2.5 (4.4)	8.6 (3.1)
17 years	55.1 (20.3)	1.3 (3.3)	238 (26.4)	2.3 (2.4)	352 (35.8)	9.9 (14)	2.2 (4.2)	8.8 (3.1)
≥18 years	54.2 (19.9)	1.2 (3.2)	226 (23.3)	2.1 (2.3)	354 (34.4)	7.9 (12.7)	1.9 (3.9)	8.9 (3.1)

*EMWSS-A* Early Memories of Warmth and Safeness Scale for Adolescents Scale (Richter et al., 2009; Portuguese version by Cunha et al., 2014), *ISSIQ-A* Impulse, Self-harm and Suicide Ideation Questionnaire for Adolescents (Carvalho et al., 2015), *ISSIQ-A Suicidal* Suicidal ideation subscale, *ISSIQ-Automatic* Automatic reinforcement subscale, *ISSIQ-A Social* Social reinforcement subscale, *STEM-B* Situational Test of Emotional Management – Brief (Allen et al., 2015; Portuguese version by da Motta et al., 2021)

^*^p < 0.05; ^**^p < 0.01; ^***^p < 0.001

Males showed significantly higher levels of early memories of warmth and safeness, automatic reinforcement function of self-harm, and social reinforcement function of self-harm than females; on the other hand, females displayed a higher prevalence of suicidal ideation and higher levels of emotion regulation than males. No significant differences were found between genders with regard to the prevalence of self-harm. Therefore, these findings only provide partial support for Hypothesis 1a—it was hypothesized that there would be no gender differences regarding both functions of self-harm and emotion regulation and, in fact, males showed higher levels of both functions of self-harm and females showed higher emotion regulation; it was hypothesized that females would display higher levels of suicidal ideation and self-harm, with the former having been confirmed and the latter not exhibiting gender differences. There were statistically significant differences between age groups with regard to all variables. Post-hoc tests revealed that 13-year-old adolescents showed higher levels of early memories of warmth and safeness than those who are 14, 15, 16, and 18 or older, with the latter displaying lower levels of the automatic and the social reinforcement functions of self-harm than most younger age groups (i.e., 13, 14, and 15 for both functions, and 16 for the automatic reinforcement function); adolescents who are 18 or older showed higher emotion regulation than 14-year-olds; 14-year-old adolescents exhibited a higher prevalence of self-harm than the remaining age groups, with 13-year-olds displaying a lower prevalence of suicidal ideation than the remaining age groups. No other significant differences between age groups were found. Therefore, these results provide support for Hypothesis 1b—that early memories of warmth and safeness and self-harm would be higher in younger adolescents and suicidal ideation and emotion regulation higher in older adolescents.

Statistically significant associations were found between all pairs of variables studied. More specifically, gender was found to be positively and weakly associated with suicidal ideation, *r* = 0.11 (*p* < 0.001), and emotion regulation, *r* = 0.20 (*p* < 0.001) – in other words, these variables were higher in females – as well as negatively and weakly associated with early memories of warmth and safeness, *r* = −0.03 (*p* = 0.026), self-harm, *r* = −0.06 (*p* < 0.001), and the automatic, *r* = −0.08 (*p* < 0.001), and the social reinforcement function of self-harm, *r* = −0.12 (*p* < 0.001) – in other words, males had higher levels of these variables. These findings provide only partial support for Hypothesis 2a – that gender would be positively associated with self-harm and suicidal ideation and would not be linked with any functions of self-harm nor emotion regulation, and, in fact, gender was only found to be positively associated with suicidal ideation and it was negatively associated with both functions of self-harm and positively associated with emotion regulation. Additionally, age was found to be positively and weakly associated with suicidal ideation, *r* = 0.04 (*p* < 0.001), and emotion regulation, *r* = 0.03 (*p* = 0.003); on the other hand, it was negatively and weakly associated with early memories of warmth and safeness, *r* = −0.03 (*p* = 0.005), self-harm, *r* = −0.03 (*p* = 0.020), and both functions, *r* = −0.08 (*p* < 0.001). These results provide support for Hypothesis 2b – that age would be negatively associated with early memories of warmth and safeness and self-harm, and positively associated with suicidal ideation and emotion regulation. Additionally, early memories of warmth and safeness were found to be positively and weakly associated with emotion regulation, *r* = 0.19 (*p* < 0.001), as well as negatively and weakly or moderately associated with the subscales of ISSIQ-A, ranging from *r* = −0.14 (*p* < 0.001) for the social reinforcement function of self-harm to *r* = −0.35 (*p* < 0.001) for suicidal ideation. Lastly, negative weak to moderate associations were found between emotion regulation and the dimensions of ISSIQ-A, ranging from *r* = −0.11 for suicidal ideation to *r* = −0.28 for the social reinforcement function of self-harm. These results provide support for Hypothesis 2c—that early emotional memories would be positively associated with emotion regulation and there would be negative associations between each of these and self-harm, its functions, and suicidal ideation. All correlation coefficients are presented in Table 3.

**Table 3:** Correlations between Gender, Age, Early Memories of Warmth and Safeness, Self-Harm, Suicidal Ideation, Automatic and Social Reinforcement Functions of Self-harm, and Emotion Regulation

Variables	1	2	3	4	5	6	7	8
1. Gender	–							
2. Age	−0.01	–						
3. EMWSS-A	−0.03^*^	−0.03^**^	–					
4. ISSIQ-A Self-harm	−0.07^***^	−0.03^*^	−0.24^***^	–				
5. ISSIQ-A Suicidal	0.11^***^	0.04^***^	−0.35^***^	0.38^***^	–			
6. ISSIQ-A Automatic	−0.08^***^	−0.08^***^	−0.17^***^	0.83^***^	0.50^***^	–		
7. ISSIQ-A Social	−0.12^***^	−0.08^***^	−0.14^***^	0.79^***^	0.41^***^	0.91^***^	–	
8. STEM-B	0.20^***^	0.03^**^	0.19^***^	−0.25^***^	−0.11^***^	−0.25^***^	−0.28^***^	–

*EMWSS-A* Early Memories of Warmth and Safeness Scale for Adolescents Scale (Richter et al., 2009; Portuguese version by Cunha et al., 2014), *ISSIQ-A* Impulse, Self-harm and Suicide Ideation Questionnaire for Adolescents (Carvalho et al., 2015), *ISSIQ-A Suicidal* Suicidal ideation subscale, *ISSIQ-Automatic* Automatic reinforcement subscale, *ISSIQ-A Social* Social reinforcement subscale, *STEM-B* Situational Test of Emotional Management – Brief (Allen et al., 2015; Portuguese version by da Motta et al., 2021)

^*^p < 0.05; ^**^p < 0.01; ^***^p < 0.001

### Moderation Analyses

**Fig. 1:**
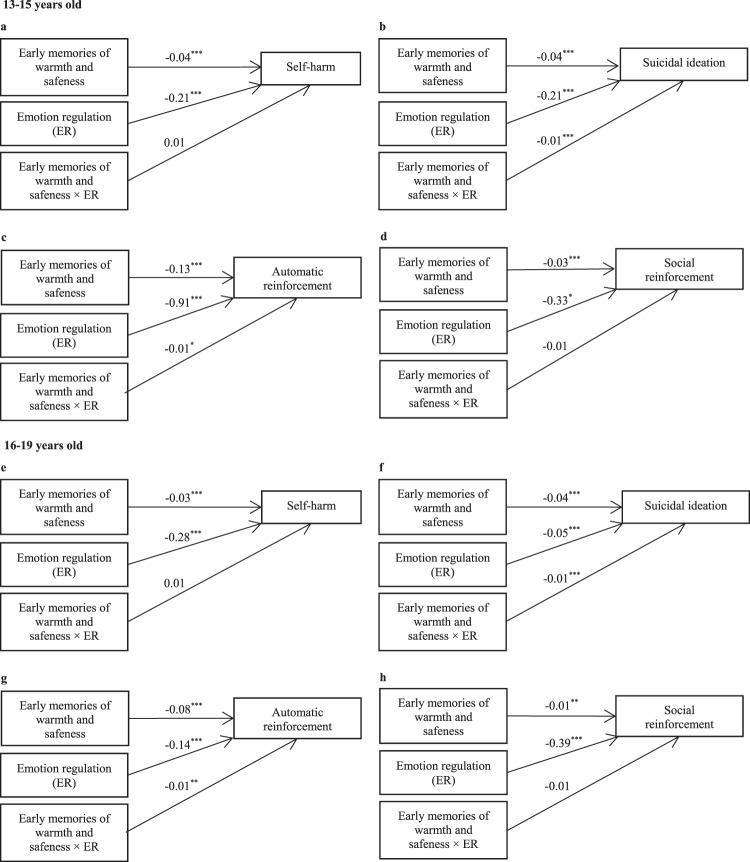
Moderated Regressions of Self-harm, Suicidal Ideation, and Automatic and Social Reinforcement Functions of Self-harm on Early Memories of Warmth and Safeness and Emotion Regulation by Age Group, controlling for Gender. Automatic reinforcement = Automatic reinforcement function of self-harm; Social reinforcement = Social reinforcement function of self-harm. All the reported parameters are unstandardized. **a** Full model *F*(4, 3376) = 103.29, *p* < 0.001; *R*^2^ = 0.109; Highest order unconditional interaction *F*(1, 3376) = 0.22, *p* = 0.638; Δ*R*^2^ = 0.001. **b** Full model *F*(4, 3746) = 162.43, *p* < 0.001; *R*^2^ = 0.148; Highest order unconditional interaction *F*(1, 3746) = 33.18, *p* < 0.001; Δ*R*^2^ = 0.008. **c** Full model *F*(4, 1724) = 34.15, *p* < 0.001; *R*^2^ = 0.073; Highest order unconditional interaction *F*(1, 1724) = 6.63, *p* = 0.010; Δ*R*^2^ = 0.004. **d** Full model *F*(4, 1724) = 36.93, *p* < 0.001; *R*^2^ = 0.079; Highest order unconditional interaction *F*(1, 1724) = 0.57, *p* = 0.450; Δ*R*^2^ = 0.001. **e** Full model *F*(4, 2945) = 80.89, *p* < 0.001; *R*^2^ = 0.099; Highest order unconditional interaction *F*(1, 2945) = 0.40, *p* = 0.526; Δ*R*^2^ = 0.001. **f** Full model *F*(4, 3174) = 145.09, *p* < 0.001; *R*^2^ = 0.155; Highest order unconditional interaction *F*(1, 3174) = 44.45, *p* < 0.001; Δ*R*^2^ = 0.012. **g** Full model *F*(4, 1487) = 40.97, *p* < 0.001; *R*^2^ = 0.099; Highest order unconditional interaction *F*(1, 1487) = 7.05, *p* = 0.008; Δ*R*^2^ = 0.004. **h** Full model *F*(4, 1487) = 48.92, *p* < 0.001; *R*^2^ = 0.116; Highest order unconditional interaction *F*(1, 1487) = 1.72, *p* = 0.190; Δ*R*^2^ = 0.001. ^*^p < 0.05; ^**^p < 0.01; ^***^p < 0.001

Four moderated regression analyses were performed using the PROCESS macro for SPSS (Hayes, 2013) by age group (i.e., 13–15, 16–19), controlling for gender given the gender differences reported above (i.e., for all variables except for the prevalence of self-harm)—resulting in a total of eight moderations – to investigate if emotion regulation acts as a moderating variable on the associations between early memories of warmth and safeness and the dimensions of the ISSIQ-A (i.e., self-harm, social and automatic reinforcement functions of self-harm, suicidal ideation), and if this moderation differs between younger and older adolescents. Considering these early memories and emotion regulation were significantly correlated, the interaction term was computed after mean-centering the independent variables (Aiken & West, 1991). The results of these regression models are summarized in Fig. 1 (see table in the Appendix for more statistical information on the regression models).

There were significant main effects of early memories of warmth and safeness, as well as emotion regulation, on self-harm, its functions, and suicidal ideation in younger (i.e., 13–15) and older (i.e., 16–19) adolescents. In both age groups, significant interaction effects were found between early memories of warmth and safeness and emotion regulation in the regression models using suicidal ideation and the automatic reinforcement function of self-harm as the outcome variables. No significant interaction effect, in both age groups, was found between early memories of warmth and safeness and emotion regulation using self-harm and its social reinforcement function as the outcome variables.

Simple slope tests were used to interpret the associations. In both younger and older adolescents, those who showed higher than average levels of this skill experienced a greater negative effect of these memories on suicidal ideation, *B* = −0.05, *p* < 0.001 in the younger adolescents, *B* = −0.06, *p* < 0.001 in those older, compared to those who exhibited average, *B* = −0.04, *p* < 0.001 in both age groups, or lower than average, *B* = −0.03, *p* < 0.001 in both age groups, values of emotion regulation. In the group of the younger adolescents, those who exhibited higher than average levels of emotion regulation experienced a greater negative effect of early memories of warmth and safeness on the automatic reinforcement function of self-harm, *B* = −0.17, *p* < 0.001, compared to those who showed average levels of emotion regulation, *B* = −0.13, *p* < 0.001, with adolescents who displayed lower than average levels of this skill not experiencing this effect, *B* = −0.08, *p* < 0.001; on the other hand, in the group of older the adolescents, at lower than average levels of emotion regulation no significant effect of early memories of warmth and safeness was found on the automatic reinforcement function of SF, *B* = −0.04, *p* = 0.078, with those who showed higher than average levels of emotion regulation experiencing a greater negative effect of early memories of warmth and safeness on this function of self-harm, *B* = −0.12, *p* < 0.001, compared to those who exhibited average levels of emotion regulation, *B* = −0.08, *p* < 0.001.

In both younger and older adolescents, these results identify emotion regulation as a significant negative moderator on the relationships between early memories of warmth and safeness and the following variables and suicidal ideation, as well as the automatic reinforcement function of self-harm. On the other hand, also in both age groups, the findings indicate that emotion regulation was not a moderator on the associations between early memories of warmth and safeness and self-harm, as well as its social reinforcement function. These results provide only partial support for Hypothesis 3 – that emotion regulation would moderate the associations between early emotional memories and the adolescent risks measured and, in fact, this only emerged for suicidal ideation and the automatic reinforcement function of self-harm.

### Sensitivity Analyses

Given that the categories of severity of suicidal ideation were relatively arbitrary (i.e., on a single item, answering 1 = *Sometimes happens to me* was considered indicative of having moderate suicidal ideation, answering 2 = *Often happens to me* indicative of severe suicidal ideation, and answering 3 = *Always happens to me* indicative of very severe suicidal ideation), sensitivity analyses were conducted regarding this severity to characterize suicidal ideation using a different cut-off point—answering 2 or 3 considered indicative of severe suicidal ideation. This new cut-off leads to a percentage of 12.4% of adolescents with severe suicidal ideation in the current sample. Additionally, considering that in the present study, a relatively arbitrary dichotomization was made with regard to age groups (i.e., younger adolescents were considered those who are 13–15 years old and older adolescents were considered those who are 16-19 years old), sensitivity analyses were conducted to explore whether the moderation results were the same for every specific age group (i.e., 13, 14, 15, 16, 17, 18 or older). See Supplementary Text, Supplementary Table, and Supplementary Figure for the moderation model results and their descriptions. The results differed to a certain degree across age groups: the findings were exactly the same only in the 15-year-olds, in other words the moderation of emotion regulation was significant for suicidal ideation and the automatic reinforcement function of self-harm; similar to the results, a non-significant moderation of emotion regulation for self-harm was found across all ages; regarding suicidal ideation, similar to the findings, the moderation was significant in adolescents who are 13, 14, 16, and 18 or older; with regard to the automatic reinforcement function of self-harm, similar to the results, the moderation was significant only in the 16-year-olds; regarding the social reinforcement function of self-harm, contrasting with the findings, the moderation was significant only in the 16-year olds.

## Discussion

Previous research shows that early memories of warmth and safeness and emotion regulation have a protective effect against self-harm and suicidal ideation in adolescence; however, no previous study has examined the possible interaction effect that the former variables have on the latter across different stages of adolescence. To address this gap, using a representative sample of adolescents living on nine islands of the Autonomous Region of the Azores (Portugal), this study explored the moderating role of emotion regulation in the associations between these early emotional memories, self-harm, both its functions (i.e., automatic and social reinforcement), and suicidal ideation in adolescence, in younger and older age groups (i.e., 13–5, 16–19). In both age groups, emotion regulation increased the negative effect of these memories on suicidal ideation and the automatic reinforcement function of self-harm.

Firstly, this study aimed to characterize these variables, including the prevalence of self-harm and suicidal ideation, in the total sample and by gender and age group (i.e., 13, 14, 15, 16, 17, 18 or older). It was hypothesized that females would show higher self-harm and suicidal ideation and that there would be no differences regarding both functions of self-harm and emotion regulation; it was also hypothesized that younger age groups (i.e., 13, 14, 15) would exhibit higher early memories of warmth and safeness and self-harm, and older age groups (i.e., 16, 17, 18 or older) higher suicidal ideation and emotion regulation. Additionally, the study aimed to examine the associations between gender, age, and all the previously mentioned variables. It was hypothesized that gender would be positively associated with self-harm and suicidal ideation, and that it would not be associated with any functions of self-harm nor emotion regulation. In addition, it was hypothesized that age would be negatively associated with early memories of warmth and safeness and self-harm, as well as positively associated with suicidal ideation and emotion regulation; it was also hypothesized that positive associations would be found between emotion regulation and early memories of warmth and safeness, and negative associations between each of these and adolescent self-harm, its functions, and suicidal ideation.

Concerningly high rates of adolescent suicidal ideation and self-harm were found in the present sample—34.5% and 28%, respectively—with the former being higher than the rates of suicidal ideation found using a Portuguese adolescent sample (Barreto Carvalho et al., 2017: 22%) and an international sample (Biswas et al., 2020: 14%), and the latter being relatively aligned with the rates of self-harm found in previous studies using adolescent Portuguese samples (Barreto Carvalho et al., 2017: 29.5%; Equipa Aventura Social, 2022: 24.6%). The automatic reinforcement function of self-harm was found to be relatively more prevalent than the social reinforcement function, which indicates that self-harm is used by adolescents to regulate emotional states, either by inducing desired ones or by alleviating unpleasant emotions, to a higher extent than to influence their social environment and is in line with the previous finding that automatic reinforcement is the most frequent function of self-harm in young people (e.g., Doyle et al., 2017) and previous biological findings on self-harm and emotion regulation systems (Brezin & Gordon, 2013). These results about the (high) rates of self-harm and suicidal ideation in the present study are aligned with the long-known notion that, as a critical developmental period (e.g., Dahl et al., 2018), adolescence involves a multitude of increased risks and vulnerabilities (e.g., Zanus et al., 2021). Males showed higher levels of early memories of warmth and safeness—which is not in line with research that found that these memories do not differ by gender (Vagos et al., 2017) nor with one study that found that they are higher in females (Tahirović & Jusić, 2016)—and higher levels of both functions of self-harm—which is not aligned with previous research that found no gender differences with regard to these functions (e.g., Calvete et al., 2015); on the other hand, females showed higher suicidal ideation—in line with previous studies (e.g., Voss et al., 2019)—and higher emotion regulation—which is not aligned with previous research that found no gender differences (Duarte et al., 2015). Lastly, no gender differences were found with regard to self-harm—with previous studies having found that it is more prevalent in females (for a meta-analysis, see Bresin & Schoenleber, 2015). These gender differences are aligned with the correlations found between these variables and gender (i.e., positive for suicidal ideation and emotion regulation, and negative for early memories of warmth and safeness and both functions of self-harm), except for self-harm (with which a negative correlation was found). Compared to the older adolescents, those younger showed higher early memories of warmth and safeness—in line with previous research (Xavier et al., 2016)—as well as lower suicidal ideation—aligned with the finding that these thoughts increase across adolescence (e.g., Voss et al., 2019). Younger adolescents showed higher self-harm and both its functions and lower emotion regulation—in line with the notion that self-harm decreases (Monto et al., 2018) and emotion regulation increases (Cracco et al., 2017) across adolescence; additionally, these findings support the notion that the imbalance between the underdeveloped brain structures linked to cognitive control (i.e., prefrontal cortex) and the fully developed regions related to socioemotional functions (i.e., limbic system) decreases throughout adolescence (e.g., Steinberg, 2008). These age differences are aligned with the associations found between these variables and adolescents’ age (i.e., negative for early memories of warmth and safeness, self-harm and both functions, and positive for suicidal ideation and emotion regulation).

As initially hypothesized, negative associations between emotion regulation and self-harm, both its functions, and suicidal ideation were found, in line with previous research (e.g., Brausch et al., 2022). Indeed, adolescents may use self-harm to regulate their emotions, which would be indicative of low levels of adaptive emotion regulation (e.g., Ford & Gómez, 2015). In turn, these low levels have been previously associated with suicidal ideation in adolescence (e.g., Quintana-Orts et al., 2020). Furthermore, as hypothesized, negative associations between early memories of warmth and safeness self-harm and suicidal ideation were found, corroborating the results of previous studies using Portuguese adolescent samples (Barreto Carvalho et al., 2015, 2017). Similarly, previous research had found that parenting qualities related to warmth were associated with a decreased risk of self-harm (Ran et al., 2021) and suicidal ideation in adolescence (e.g., Gorostiaga et al., 2019). Lastly, positive associations between early emotional memories and emotion regulation were found, as hypothesized, in line with previous findings that these memories are linked with indicators of adaptive emotion regulation (e.g., Marta-Simões & Ferreira, 2020).

In younger (i.e., 13–15) and older (i.e., 16–19) adolescents, emotion regulation was not found to be a significant moderator in the association between early memories of warmth and safeness and adolescent self-harm; this may be explained due to the likely possibility that adolescents who reported self-injuring behaviors use it for additional purposes besides regulating their own emotions (i.e., automatic reinforcement function) such as to avoid interpersonal conflict or to receive attention from others (i.e., social reinforcement function). It is also likely that one portion of adolescents engages in self-harm at least partially due to social modeling/learning as a result of exposure to and/or contact with peers who also engage in this behavior, with the social contagion effect of self-harm in youth having been well documented (e.g., Jarvi et al., 2013); it is likely that in insular regions—such as the Azores, where the sample is from—in which social and geographical proximity is higher, this effect is particularly prevalent. Additionally, also in both age groups, emotion regulation was not a moderator in the association between early emotional memories and the social reinforcement function of self-harm; this finding may be explained by the likely possibility that this function of self-harm is less linked to emotional childhood memories and emotion regulation itself—compared to the automatic reinforcement function, which is directly linked to emotional states and their regulation—so it is possible that some adolescents high in these early memories (regardless of levels of emotion regulation) engage in self-harm for interpersonal reasons not linked to their primary caregivers and the emotional memories toward them (e.g., in peer or intimate relationships). Taken together, these results suggest that, compared to other functions of adolescent self-harm, the risk for the automatic reinforcement function is particularly decreased by high levels of emotion regulation. Indeed, in both younger and older adolescents, this skill was found to be a moderator in the associations between early memories of warmth and safeness and the automatic reinforcement function of self-harm, as well as between these memories and suicidal ideation. In other words, higher levels of emotion regulation were found to increase the strength of the negative associations between these early emotional memories and the previously mentioned risk-related outcomes. These results indicate that emotion regulation and early memories of warmth and safeness have a combined protective role against the occurrence of these risks and vulnerabilities in adolescence. However, specifically in older adolescents with low emotion regulation, these early memories did not have a negative effect (i.e., a protective effect) on the automatic reinforcement function of self-harm—whereas this effect was found in younger adolescents also with low emotion regulation. It is expected that throughout adolescence, individuals go through a critical process of identity formation that often involves an increasing “separation” from their primary caregivers (e.g., family) (Erikson, 1950, 1968; Marcia, 1966), which in turn may be associated with a lower regard for memories linked to one’s family (e.g., early memories of warmth and safeness) and a higher reliance on individual characteristics (e.g., emotion regulation) toward a higher sense of independence; older adolescents—which according to a normative identity development are expected to have a higher sense of self and autonomy from their families, possibly leading to a lower regard for family memories – with low emotion regulation are more prone to engage in risk-behaviors such as self-harm. In both age groups, regarding the functions of self-harm, emotion regulation only moderated the association between early memories of warmth and safeness and the automatic reinforcement function (and not the social reinforcement function); this finding has important practical implications in that it is relevant to identify the function/s that self-harm serve/s when intervening with younger or older adolescents who engage in this behavior, with targeting emotion regulation being particularly relevant if the primary function of self-harm is the regulation of emotional states.

The results point to the importance of promoting emotion regulation across the whole adolescent developmental period (both in younger and older adolescents) and across different contexts (e.g., family, school) to prevent or mitigate the risk of suicidal ideation and adolescent engagement in self-injuring behaviors, particularly when these serve the purpose of regulating one’s emotional states, regardless of levels of early memories of warmth and safeness. For instance, the teaching of adaptive emotion regulation strategies—and other emotion-related skills such as emotional intelligence—should be prioritized in educational curricula across multiple school years (e.g., 6th–12th grade) and the creation of emotionally intelligent environments (e.g., with the help of specialized training) in institutions and other entities that serve adolescents (e.g., recreation centers, sport clubs) should also be considered. Considering the role that the family environment and the associated early emotional memories have on self-harm (e.g., Ran et al., 2021) and suicidal ideation (e.g., Gorostiaga et al., 2019) in adolescence, it is also essential to promote emotion regulation skills in caregivers, so that they are then more capable of providing education and care to the children/adolescents they care for, as well as teach them how to regulate their own emotions in an adaptive manner and within an emotionally intelligent environment. For example, emotion regulation skills should be a focus of interventions and programs promoting positive parenting practices and behaviors.

This study has some limitations, including its cross-sectional design, which does not allow for the accurate inference of predictive relationships between variables over time; the use of self-report measures and one retrospective measure, which may have a negative impact on the results; and the lengthiness of the full research protocol used, which may induce participant fatigue (and in turn bias the results), even though adolescent participation was split into two different moments. Future studies should extend the findings of this research by examining whether emotion regulation—as well as whether different adaptive emotion regulation strategies (e.g., acceptance, positive reappraisal, refocus on planning)—also acts as a moderator in the associations between early memories of warmth and safeness and other risk-related outcomes in adolescence (e.g., high-risk behaviors), as well as in the links between other variables related to the family environment and early experiences (e.g., adverse childhood experiences, attachment style), and adolescent self-harm, suicidal ideation, and other risk-related outcomes. Future research should also use longitudinal designs to further examine the effect of emotion regulation in the associations between these early emotional memories (and other family-related variables) and risk-related outcomes in adolescence (e.g., self-harm, suicidal ideation) across different time periods and different developmental stages (e.g., childhood, 10–12-year-old adolescents). Additionally, future quasi-experimental studies should also examine the effect of interventions and programs targeting emotion regulation in the relationships between early emotional memories and these vulnerabilities in adolescence; it would be reasonable to hypothesize that the effect would be stronger in the experimental group compared to adolescent community samples (i.e., control groups). Lastly, the role of caregiver emotion regulation in these associations should also be explored.

## Conclusion

Previous research had found that self-harm and suicidal ideation in adolescence are negatively linked with early memories of warmth and safeness and emotion regulation, with the latter two having been positively associated (with one another). Using a representative sample of adolescents living on nine islands of the Autonomous Region of the Azores, Portugal, this study extends prior works by exploring the moderating role of emotion regulation in the associations between early memories of warmth and safeness, and the above-mentioned vulnerabilities, in both younger (i.e., 13–15) and older (i.e., 16–19) adolescents. In both age groups, emotion regulation was found to enhance the (negative) associations between these early emotional memories and the automatic reinforcement function of self-harm and suicidal ideation. These results highlight the importance of promoting emotion regulation, across the whole adolescent developmental period (both in younger and older adolescents) and across several contexts (e.g., family, school), for the prevention or mitigation of suicidal ideation and self-harm in adolescence, especially when the latter is used to create desirable emotional states or alleviate unpleasant emotions, regardless of levels of early memories of warmth and safeness.

### Supplementary Information

Supplementary material

## Appendix

Moderated Regressions of Self-harm, Suicidal Ideation, and Automatic and Social Reinforcement Functions of Self-harm on Early Memories of Warmth and Safeness and Emotion Regulation by Age Group, controlling for Gender

**Table:**

Regression models	*B*	95% CI		*t*	*p*	*F*	*p*	*R*^2^
		*LL*	*UL*					
13–15 years old								
DV: ISSIQ-A Self-harm						103.29	<0.001	0.109
EMWSS-A	−0.04	−0.05	−0.04	−14.78	<0.001			
STEM-B	−0.21	−0.25	−0.17	−10.19	<0.001			
EMWSS-A × STEM-B	0.01	−0.01	0.01	0.47	0.638			
Δ*R*^2^ = 0.001						0.22	0.638	
DV: ISSIQ-A Suicidal						162.43	<0.001	0.148
EMWSS-A	−0.04	−0.05	−0.04	−22.31	<0.001			
STEM-B	−0.07	−0.09	−0.04	−5.06	<0.001			
EMWSS-A × STEM-B	−0.01	−0.01	−0.01	−5.76	<0.001			
Δ*R*^2^ = 0.008						33.18	<0.001	
DV: ISSIQ-A Automatic						34.15	<0.001	0.073
EMWSS-A	−0.13	−0.16	−0.09	−7.23	<0.001			
STEM-B	−0.91	−1.15	−0.67	−7.33	<0.001			
EMWSS-A × STEM-B	−0.01	−0.02	−0.01	−2.57	0.010			
Δ*R*^2^ = 0.004						6.63	0.010	
DV: ISSIQ-A Social						36.93	<0.001	0.079
EMWSS-A	−0.03	−0.04	−0.02	−5.79	<0.001			
STEM-B	−0.33	−0.40	−0.26	−8.87	<0.001			
EMWSS-A × STEM-B	−0.01	−0.01	0.01	−0.76	0.450			
Δ*R*^2^ = 0.001						0.57	0.450	
16–19 years old								
DV: ISSIQ-A Self-harm						80.89	<0.001	0.099
EMWSS-A	−0.03	−0.03	−0.02	−8.21	<0.001			
STEM-B	−0.28	−0.32	−0.24	−13.33	<0.001			
EMWSS-A × STEM-B	0.01	−0.01	0.01	−0.63	0.526			
Δ*R*^2^ = 0.001						0.40	0.526	
DV: ISSIQ-A Suicidal						145.09	<0.001	0.155
EMWSS-A	−0.04	−0.05	−0.04	−21.55	<0.001			
STEM-B	−0.05	−0.07	−0.02	−3.44	<0.001			
EMWSS-A × STEM-B	−0.01	−0.01	−0.01	−6.67	<0.001			
Δ*R*^2^ = 0.012						44.45	<0.001	
DV: ISSIQ-A Automatic						40.97	<0.001	0.099
EMWSS-A	−0.08	−0.11	−0.05	−4.74	<0.001			
STEM-B	−1.14	−1.36	−0.91	−9.87	<0.001			
EMWSS-A × STEM-B	−0.01	−0.02	−0.01	−2.66	0.008			
Δ*R*^2^ = 0.004						7.05	0.008	
DV: ISSIQ-A Social						48.92	<0.001	0.116
EMWSS-A	−0.01	−0.02	−0.01	−2.75	0.006			
STEM-B	−0.39	−0.46	−0.33	−11.54	<0.001			
EMWSS-A × STEM-B	−0.01	−0.01	0.01	−1.31	0.190			
Δ*R*^2^ = 0.001						1.72	0.190	

*DV* dependent variable, *EMWSS-A* Early Memories of Warmth and Safeness Scale for Adolescents Scale (Richter et al., 2009; Portuguese version by Cunha et al., 2014); *ISSIQ-A* Impulse, Self-harm and Suicide Ideation Questionnaire for Adolescents (Carvalho et al., 2015), *ISSIQ-A Suicidal* Suicidal ideation subscale, *ISSIQ-Automatic* Automatic reinforcement subscale, *ISSIQ-A Social* Social reinforcement subscale, *STEM-B* Situational Test of Emotional Management—Brief (Allen et al., 2015; Portuguese version by da Motta et al., 2021). *CI* confidence interval, *LL* lower limit, *UL* upper limit.
