# Supplementary material for: The Enhancing Role of Emotion Regulation in the Links between Early Positive Memories and Self-harm and Suicidal Ideation in Adolescence
Source: J Youth Adolesc. 2023 May 13;52(8):1738–52. doi: 10.1007/s10964-023-01777-8 (PMC10275796; doi:10.1007/s10964-023-01777-8)
Supplement: Supplementary file 1 — Supplementary material [file 10964_2023_1777_MOESM1_ESM.docx]

**Supplementary Material**

Supplementary Text. Description of Sensitivity Analyses

Supplementary Table. Moderated Regressions of Self-Harm, Suicidal Ideation, and Automatic and Social Reinforcement Functions of Self-harm on Early Memories of Warmth and Safeness and Emotion Regulation by Age, controlling for Gender

Supplementary Figure. Moderated Regressions of Self-harm, Suicidal Ideation, and Automatic and Social Reinforcement Functions of Self-harm on Early Memories of Warmth and Safeness and Emotion Regulation by Age, controlling for Gender

Supplementary Text. Description of Sensitivity Analyses

Four moderated regression analyses were performed using the PROCESS macro for SPSS (Hayes, 2013) by age (i.e., 13, 14, 15, 16, 17, 18 or older), controlling for gender – resulting in a total of 24 moderations – to investigate if emotion regulation acts as a moderating variable on the associations between early memories of warmth and safeness and the dimensions of the ISSIQ-A (i.e., SH, social and automatic reinforcement functions of SH, suicidal ideation). Considering these early memories and emotion regulation were significantly correlated, the interaction term was computed after mean-centering the independent variables (Aiken & West, 1991).

There were significant main effects of early memories of warmth and safeness on SH and suicidal ideation across all age groups, as well as on the automatic and the social reinforcement functions of SH across all age groups except for the group of 16-year-old adolescents. There were also significant main effects of emotion regulation on SH and its social reinforcement function across all age groups, as well as on suicidal ideation and on the automatic reinforcement function of SH in the groups of adolescents who are 14, 15, 16, and 17 years old, with emotion regulation also showing a main effect on the latter (i.e., automatic reinforcement function of SH) in those above the age of 18. Significant interaction effects were found between early memories of warmth and safeness and emotion regulation in the regression models using the following dimensions of the ISSIQ-A as outcome variables: suicidal ideation, across all age groups except for those who are 17 years old; the automatic reinforcement function of SH, in the groups of the 15-year-old and 16-year-old adolescents; and the social reinforcement function of SH, only in the group of those who are 16 years old. No significant interaction effect, across any age groups, was found between early memories of warmth and safeness and emotion regulation using SH as the outcome variable.

Simple slopes tests were used to interpret the associations. Regarding suicidal ideation, in the age groups where a significant interaction effect was found between early memories of warmth and safeness and emotion regulation (i.e., 13, 14, 15, 16, 18 or older), the adolescents who showed higher than average levels of this skill experienced a greater effect of these memories on suicidal ideation – *B* ranging from -0.05, *p* < .001, in the 13 and 14-year-old adolescents to -0.06, *p* < .001, in those who are 15, 16, and 18 or older – compared to those who exhibited average – *B* = -0.04, *p* < .001, across all these age groups – or lower than average – *B* ranging from -0.02, *p* < .001, in the 16-year-old adolescents to -0.04, *p* < .001, in those who are 13 – values of emotion regulation. In relation to the automatic reinforcement function of SH: in the group of the 15-year-old adolescents, those who exhibited higher than average levels of emotion regulation experienced a greater negative effect of early memories of warmth and safeness on the automatic reinforcement function of SH, *B* = -0.16, *p* < .001, compared to those who showed average levels of emotion regulation, *B* = -0.10, *p* < .001, with adolescents who displayed lower than average levels of this skill not experiencing this effect, *B* = -0.03, *p* = .362; in the group of the 16-year-old adolescents, a negative effect of early memories of warmth and safeness on the automatic reinforcement function of SH was only found in the those who showed higher than average levels of emotion regulation, *B* = -0.11, *p* = .012. Lastly, regarding the social reinforcement function of SH, despite the fact that a significant interaction effect was found between early memories of warmth and safeness and emotion regulation, there was no effect of the former on the social reinforcement function of SH at any levels (i.e., lower than average, average, higher than average) of emotion regulation.

These results identify emotion regulation as a significant negative moderator on the relationships between early memories of warmth and safeness and the following variables: 1) suicidal ideation, in the ages of 13, 14, 15, 16, and 18 or older; 2) automatic reinforcement function of SH, in the ages of 15 and 16; 3) social reinforcement function of SH, in the group of the 16-year-old adolescents (although no effects were found of early memories of warmth and safeness at any levels of emotion regulation). On the other hand, the findings indicate that emotion regulation was not a moderator on the associations between early memories of warmth and safeness and SH, across any age groups.

Supplementary Table. Moderated Regressions of Self-Harm, Suicidal Ideation, and Automatic and Social Reinforcement Functions of Self-harm on Early Memories of Warmth and Safeness and Emotion Regulation by Age, controlling for Gender

| Regression models | *B* | 95% CI | | *t* | *p* | *F* | *p* | *R*^2^ |
| --- | --- | --- | --- | --- | --- | --- | --- | --- |
|  |  | *LL* | *UL* |  |  |  |  |  |
| 13-year-olds |  |  |  |  |  |  |  |  |
| DV: ISSIQ-A Self-harm |  |  |  |  |  | 36.94 | < .001 | .142 |
| EMWSS-A | -0.05 | -0.06 | -0.04 | -9.84 | < .001 |  |  |  |
| STEM-B | -0.15 | -0.23 | -0.08 | -4.03 | < .001 |  |  |  |
| EMWSS-A × STEM-B | 0.01 | -0.01 | 0.01 | 1.19 | .235 |  |  |  |
| Δ*R*^2^ = .001 |  |  |  |  |  | 01.41 | .235 |  |
| DV: ISSIQ-A Suicidal |  |  |  |  |  | 45.25 | < .001 | .152 |
| EMWSS-A | -0.04 | -0.05 | -0.04 | -12.53 | < .001 |  |  |  |
| STEM-B | -0.03 | -0.08 | 0.02 | -1.26 | .208 |  |  |  |
| EMWSS-A × STEM-B | -0.01 | -0.01 | -0.01 | -2.69 | .007 |  |  |  |
| Δ*R*^2^ = .006 |  |  |  |  |  | 7.25 | .007 |  |
| DV: ISSIQ-A Automatic |  |  |  |  |  | 11.20 | < .001 | .087 |
| EMWSS-A | -0.17 | -0.24 | -0.11 | -5.44 | < .001 |  |  |  |
| STEM-B | -0.53 | -1.00 | -0.05 | -2.19 | .029 |  |  |  |
| EMWSS-A × STEM-B | -0.01 | -0.03 | 0.01 | -0.95 | .345 |  |  |  |
| Δ*R*^2^ = .002 |  |  |  |  |  | 0.89 | .345 |  |
| DV: ISSIQ-A Social |  |  |  |  |  | 11.48 | < .001 | .089 |
| EMWSS-A | -0.05 | -0.07 | -0.03 | -4.92 | < .001 |  |  |  |
| STEM-B | -0.15 | -0.29 | -0.01 | -1.99 | .047 |  |  |  |
| EMWSS-A × STEM-B | -0.01 | -0.01 | 0.01 | -0.26 | .793 |  |  |  |
| Δ*R*^2^ = .001 |  |  |  |  |  | 0.07 | .793 |  |
| 14-year-olds |  |  |  |  |  |  |  |  |
| DV: ISSIQ-A Self-harm |  |  |  |  |  | 85.92 | < .001 | .116 |
| EMWSS-A | -0.04 | -0.05 | -0.03 | -7.66 | < .001 |  |  |  |
| STEM-B | -0.25 | -0.32 | -0.18 | -7.10 | < .001 |  |  |  |
| EMWSS-A × STEM-B | 0.01 | -0.01 | 0.01 | -0.81 | .418 |  |  |  |
| Δ*R*^2^ = .001 |  |  |  |  |  | 0.66 | .418 |  |
| DV: ISSIQ-A Suicidal |  |  |  |  |  | 47.21 | < .001 | .134 |
| EMWSS-A | -0.04 | -0.04 | -0.03 | -11.16 | < .001 |  |  |  |
| STEM-B | -0.09 | -0.13 | -0.04 | -3.77 | < .001 |  |  |  |
| EMWSS-A × STEM-B | -0.01 | -0.01 | -0.01 | -2.73 | .006 |  |  |  |
| Δ*R*^2^ = .005 |  |  |  |  |  | 7.48 | .006 |  |
| DV: ISSIQ-A Automatic |  |  |  |  |  | 11.91 | < .001 | .077 |
| EMWSS-A | -0.13 | -0.18 | -0.07 | -4.27 | < .001 |  |  |  |
| STEM-B | -0.95 | -1.35 | -0.55 | -4.65 | < .001 |  |  |  |
| EMWSS-A × STEM-B | -0.01 | -0.03 | 0.01 | -1.08 | .279 |  |  |  |
| Δ*R*^2^ = .002 |  |  |  |  |  | 1.18 | .279 |  |
| DV: ISSIQ-A Social |  |  |  |  |  | 14.31 | < .001 | .091 |
| EMWSS-A | -0.03 | -0.05 | -0.01 | -3.24 | .001 |  |  |  |
| STEM-B | -0.37 | -0.49 | -0.25 | -5.99 | < .001 |  |  |  |
| EMWSS-A × STEM-B | 0.01 | -0.01 | 0.01 | 0.31 | .754 |  |  |  |
| Δ*R*^2^ = .001 |  |  |  |  |  | 0.10 | .754 |  |
| 15-year-olds |  |  |  |  |  |  |  |  |
| DV: ISSIQ-A Self-harm |  |  |  |  |  | 34.79 | < .001 | .092 |
| EMWSS-A | -0.04 | -0.05 | -0.03 | -8.43 | < .001 |  |  |  |
| STEM-B | -0.20 | -0.27 | -0.14 | -6.14 | < .001 |  |  |  |
| EMWSS-A × STEM-B | -0.01 | -0.01 | 0.01 | -0.91 | .361 |  |  |  |
| Δ*R*^2^ = .001 |  |  |  |  |  | 0.83 | .361 |  |
| DV: ISSIQ-A Suicidal |  |  |  |  |  | 71.34 | < .001 | .159 |
| EMWSS-A | -0.04 | -0.05 | -0.04 | -14.77 | < .001 |  |  |  |
| STEM-B | -0.07 | -0.11 | -0.03 | -3.53 | < .001 |  |  |  |
| EMWSS-A × STEM-B | -0.01 | -0.01 | -0.01 | -4.29 | < .001 |  |  |  |
| Δ*R*^2^ = .010 |  |  |  |  |  | 18.40 | < .001 |  |
| DV: ISSIQ-A Automatic |  |  |  |  |  | 13.70 | < .001 | .076 |
| EMWSS-A | -0.10 | -0.15 | -0.04 | -3.39 | < .001 |  |  |  |
| STEM-B | -1.14 | -1.54 | -0.74 | -5.58 | < .001 |  |  |  |
| EMWSS-A × STEM-B | -0.02 | -0.04 | -0.01 | -2.30 | .022 |  |  |  |
| Δ*R*^2^ = .007 |  |  |  |  |  | 5.30 | .022 |  |
| DV: ISSIQ-A Social |  |  |  |  |  | 15.14 | < .001 | .083 |
| EMWSS-A | -0.02 | -0.04 | -0.01 | -2.39 | .017 |  |  |  |
| STEM-B | -0.40 | -0.52 | -0.28 | -6.60 | < .001 |  |  |  |
| EMWSS-A × STEM-B | -0.01 | -0.01 | 0.01 | -1.32 | .187 |  |  |  |
| Δ*R*^2^ = .002 |  |  |  |  |  | 1.74 | .187 |  |
| 16-year-olds |  |  |  |  |  |  |  |  |
| DV: ISSIQ-A Self-harm |  |  |  |  |  | 32.07 | < .001 | .103 |
| EMWSS-A | -0.02 | -0.03 | -0.01 | -4.25 | < .001 |  |  |  |
| STEM-B | -0.33 | -0.40 | -0-26 | -8.95 | < .001 |  |  |  |
| EMWSS-A × STEM-B | -0.01 | -0.01 | 0.01 | -0.76 | .448 |  |  |  |
| Δ*R*^2^ = .001 |  |  |  |  |  | 0.58 | .448 |  |
| DV: ISSIQ-A Suicidal |  |  |  |  |  | 48.77 | < .001 | .139 |
| EMWSS-A | -0.04 | -0.04 | -0.03 | -11.40 | < .001 |  |  |  |
| STEM-B | -0.07 | -0.11 | -0.02 | -2.87 | .004 |  |  |  |
| EMWSS-A × STEM-B | -0.01 | -0.01 | -0.01 | -6.15 | < .001 |  |  |  |
| Δ*R*^2^ = .027 |  |  |  |  |  | 37.81 | < .001 |  |
| DV: ISSIQ-A Automatic |  |  |  |  |  | 17.68 | < .001 | .108 |
| EMWSS-A | -0.03 | -0.09 | 0.02 | -1.14 | .256 |  |  |  |
| STEM-B | -1.40 | -1.80 | -1 | -6.88 | < .001 |  |  |  |
| EMWSS-A × STEM-B | -0.03 | -0.04 | -0.01 | -2.77 | .006 |  |  |  |
| Δ*R*^2^ = .012 |  |  |  |  |  | 7.65 | .006 |  |
| DV: ISSIQ-A Social |  |  |  |  |  | 21.46 | < .001 | .128 |
| EMWSS-A | -0.01 | -0.02 | 0.02 | -0.02 | .986 |  |  |  |
| STEM-B | -0.45 | -0.57 | -0.33 | -7.51 | < .001 |  |  |  |
| EMWSS-A × STEM-B | -0.01 | -0.01 | -0.01 | -2.03 | .043 |  |  |  |
| Δ*R*^2^ = .006 |  |  |  |  |  | 4.13 | .043 |  |
| 17-year-olds |  |  |  |  |  |  |  |  |
| DV: ISSIQ-A Self-harm |  |  |  |  |  | 22.12 | < .001 | .092 |
| EMWSS-A | -0.02 | -0.03 | -0.01 | -3.99 | < .001 |  |  |  |
| STEM-B | -0.27 | -0.34 | -0.20 | -7.41 | < .001 |  |  |  |
| EMWSS-A × STEM-B | -0.01 | -0.01 | 0.01 | -0.23 | .818 |  |  |  |
| Δ*R*^2^ = .001 |  |  |  |  |  | 0.05 | .818 |  |
| DV: ISSIQ-A Suicidal |  |  |  |  |  | 62.03 | < .001 | .206 |
| EMWSS-A | -0.05 | -0.06 | -0.04 | -13.99 | < .001 |  |  |  |
| STEM-B | -0.07 | -0.11 | -0.02 | -2.97 | .003 |  |  |  |
| EMWSS-A × STEM-B | -0.01 | -0.01 | 0.01 | -1.14 | .254 |  |  |  |
| Δ*R*^2^ = .001 |  |  |  |  |  | 1.30 | .254 |  |
| DV: ISSIQ-A Automatic |  |  |  |  |  | 16.91 | < .001 | .134 |
| EMWSS-A | -0.11 | -0.17 | -0.06 | -3.99 | < .001 |  |  |  |
| STEM-B | -1.10 | -1.49 | -0.71 | -5.57 | < .001 |  |  |  |
| EMWSS-A × STEM-B | -0.02 | -0.03 | 0.01 | -1.92 | .055 |  |  |  |
| Δ*R*^2^ = .007 |  |  |  |  |  | 3.70 | .055 |  |
| DV: ISSIQ-A Social |  |  |  |  |  | 18.08 | < .001 | .142 |
| EMWSS-A | -0.02 | -0.04 | -0.01 | -2.46 | .014 |  |  |  |
| STEM-B | -0.39 | -0.51 | -0.27 | -6.58 | < .001 |  |  |  |
| EMWSS-A × STEM-B | -0.01 | -0.01 | 0.01 | -0.79 | .433 |  |  |  |
| Δ*R*^2^ = .001 |  |  |  |  |  | 0.62 | .433 |  |
| 18 or older |  |  |  |  |  |  |  |  |
| DV: ISSIQ-A Self-harm |  |  |  |  |  | 18.13 | < .001 | .098 |
| EMWSS-A | -0.03 | -0.04 | -0.02 | -5.80 | < .001 |  |  |  |
| STEM-B | -0.16 | -0.23 | -0.09 | -4.29 | < .001 |  |  |  |
| EMWSS-A × STEM-B | -0.01 | -0.01 | 0.01 | -0.94 | .349 |  |  |  |
| Δ*R*^2^ = .001 |  |  |  |  |  | 0.88 | .349 |  |
| DV: ISSIQ-A Suicidal |  |  |  |  |  | 32.62 | < .001 | .156 |
| EMWSS-A | -0.04 | -0.05 | -0.04 | -10.81 | < .001 |  |  |  |
| STEM-B | 0.03 | -0.03 | 0.08 | -0.97 | .333 |  |  |  |
| EMWSS-A × STEM-B | -0.01 | -0.01 | -0.01 | -4.33 | < .001 |  |  |  |
| Δ*R*^2^ = .022 |  |  |  |  |  | 18.71 | < .001 |  |
| DV: ISSIQ-A Automatic |  |  |  |  |  | 7.39 | < .001 | .087 |
| EMWSS-A | -0.12 | -0.18 | -0.06 | -4.18 | < .001 |  |  |  |
| STEM-B | -0.82 | -1.18 | -0.46 | -4.43 | < .001 |  |  |  |
| EMWSS-A × STEM-B | -0.01 | -0.02 | 0.02 | -0.06 | .948 |  |  |  |
| Δ*R*^2^ = .001 |  |  |  |  |  | 0.01 | .948 |  |
| DV: ISSIQ-A Social |  |  |  |  |  | 9.84 | < .001 | .112 |
| EMWSS-A | -0.03 | -0.05 | -0.01 | -3.39 | .001 |  |  |  |
| STEM-B | -0.21 | -0.33 | -0.09 | -3.48 | .001 |  |  |  |
| EMWSS-A × STEM-B | 0.01 | -0.01 | 0.01 | 1.16 | .249 |  |  |  |
| Δ*R*^2^ = .004 |  |  |  |  |  | 1.34 | .249 |  |

*Note*. DV = Dependent variable; EMWSS-A = Early Memories of Warmth and Safeness Scale for Adolescents Scale (Richter et al., 2009; Portuguese version by Cunha et al., 2014); ISSIQ-A = Impulse, Self-harm and Suicide Ideation Questionnaire for Adolescents (Carvalho et al., 2015); ISSIQ-A Suicidal = Suicidal ideation subscale; ISSIQ-Automatic = Automatic reinforcement subscale; ISSIQ-A Social = Social reinforcement subscale; STEM-B = Situational Test of Emotional Management – Brief (Allen et al., 2015; Portuguese version by da Motta et al., 2021). CI = confidence interval; *LL* = lower limit; *UL* = upper limit.

Supplementary Figure. Moderated Regressions of Self-harm, Suicidal Ideation, and Automatic and Social Reinforcement Functions of Self-harm on Early Memories of Warmth and Safeness and Emotion Regulation by Age, controlling for Gender

**b**

**a**

**13-year-olds**

**c**

**d**

**f**

**e**

**14-year-olds**

**g**

**h**

**j**

**i**

**15-year-olds**

Early memories of warmth and safeness

-0.04^***^

-0.03

Suicidal ideation

-0.01^**^

Emotion regulation (ER)

Early memories of warmth and safeness × ER

Self-harm

Early memories of warmth and safeness

-0.05^***^

-0.15^***^

0.01

Emotion regulation (ER)

Early memories of warmth and safeness × ER

-0.01

Early memories of warmth and safeness

-0.05^***^

Social

reinforcement

-0.15^*^

Emotion regulation (ER)

Early memories of warmth and safeness × ER

0.01

Early memories of warmth and safeness

-0.03^**^

Social

reinforcement

-0.37^***^

Emotion regulation (ER)

Early memories of warmth and safeness × ER

Self-harm

Early memories of warmth and safeness

-0.04^***^

-0.20^***^

-0.01

Emotion regulation (ER)

Early memories of warmth and safeness × ER

Early memories of warmth and safeness

-0.04^***^

-0.07^***^

Suicidal ideation

-0.01^***^

Emotion regulation (ER)

Early memories of warmth and safeness × ER

-0.53^*^

Early memories of warmth and safeness

-0.13^***^

Automatic

reinforcement

Emotion regulation (ER)

Early memories of warmth and safeness × ER

-0.01

-0.95^***^

Self-harm

Early memories of warmth and safeness

-0.04^***^

-0.25^***^

0.01

Emotion regulation (ER)

Early memories of warmth and safeness × ER

Early memories of warmth and safeness

-0.04^***^

-0.09^***^

Suicidal ideation

-0.01^**^

Emotion regulation (ER)

Early memories of warmth and safeness × ER

Early memories of warmth and safeness

-0.17^***^

Automatic

reinforcement

Emotion regulation (ER)

Early memories of warmth and safeness × ER

-0.01

**l**

**k**

**n**

**m**

**16-year-olds**

**o**

**p**

**r**

**q**

**17-year-olds**

**s**

**t**

Early memories of warmth and safeness

-0.10^***^

Automatic

reinforcement

Emotion regulation (ER)

Early memories of warmth and safeness × ER

-0.02^*^

-1.14^***^

Early memories of warmth and safeness

-0.02^*^

Social

reinforcement

-0.40^***^

Emotion regulation (ER)

Early memories of warmth and safeness × ER

-0.01

Self-harm

Early memories of warmth and safeness

-0.02^***^

-0.33^***^

-0.01

Emotion regulation (ER)

Early memories of warmth and safeness × ER

Early memories of warmth and safeness

-0.04^***^

-0.07^**^

Suicidal ideation

-0.01^***^

Emotion regulation (ER)

Early memories of warmth and safeness × ER

Early memories of warmth and safeness

-0.01

Social

reinforcement

-0.45^***^

Emotion regulation (ER)

Early memories of warmth and safeness × ER

-0.01^*^

Early memories of warmth and safeness

-0.03

Automatic

reinforcement

Emotion regulation (ER)

Early memories of warmth and safeness × ER

-0.03^**^

-1.40^***^

Self-harm

Early memories of warmth and safeness

-0.02^***^

-0.27^***^

-0.01

Emotion regulation (ER)

Early memories of warmth and safeness × ER

Early memories of warmth and safeness

-0.05^***^

-0.07^**^

Suicidal ideation

-0.01

Emotion regulation (ER)

Early memories of warmth and safeness × ER

Early memories of warmth and safeness

-0.02^*^

Social

reinforcement

-0.39^***^

Emotion regulation (ER)

Early memories of warmth and safeness × ER

-0.01

Early memories of warmth and safeness

-0.11^***^

Automatic

reinforcement

Emotion regulation (ER)

Early memories of warmth and safeness × ER

-0.02

-1.10^***^

**v**

**u**

-0.01

**18 or older**

**w**

**x**

Early memories of warmth and safeness

-0.04^***^

0.03

Suicidal ideation

-0.01^***^

Emotion regulation (ER)

Early memories of warmth and safeness × ER

Self-harm

Early memories of warmth and safeness

-0.03^***^

-0.16^***^

Emotion regulation (ER)

Early memories of warmth and safeness × ER

Early memories of warmth and safeness

-0.12^***^

Automatic

reinforcement

Emotion regulation (ER)

Early memories of warmth and safeness × ER

-0.01

-0.82^***^

Early memories of warmth and safeness

-0.03^**^

Social

reinforcement

-0.21^**^

Emotion regulation (ER)

Early memories of warmth and safeness × ER

0.01

*Note*. Automatic reinforcement = Automatic reinforcement function of self-harm; Social reinforcement = Social reinforcement function of self-harm. All the reported parameters are unstandardized.

a: Full model *F*(4, 894) = 36.94, *p* < .001; *R*^2^ = .142; Highest order unconditional interaction *F*(1, 894) = 1.41, *p* = .235; Δ*R*^2^ = .001

b: Full model *F*(4, 1010) = 45.25, *p* < .001; *R*^2^ = .152; Highest order unconditional interaction *F*(1, 1010) = 7.25, *p* = .007; Δ*R*^2^ = .006

c: Full model *F*(4, 472) = 11.20, *p* < .001; *R*^2^ = .087; Highest order unconditional interaction *F*(1, 472) = 0.89, *p* =.345; Δ*R*^2^ = .002

d: Full model *F*(4, 472) = 11.48, *p* < .001; *R*^2^ = .089; Highest order unconditional interaction *F*(1, 472) = 0.07, *p* =.793; Δ*R*^2^ = .001

e: Full model *F*(4, 1094) = 35.92, *p* < .001; *R*^2^ = .116; Highest order unconditional interaction *F*(1, 1094) = 0.66, *p* = .418; Δ*R*^2^ = .001

f: Full model *F*(4, 1220) = 47.21, *p* < .001; *R*^2^ = .134; Highest order unconditional interaction *F*(1, 1220) = 7.48, *p* = .006; Δ*R*^2^ = .005

g: Full model *F*(4, 573) = 11.91, *p* < .001; *R*^2^ = .077; Highest order unconditional interaction *F*(1, 573) = 1.18, *p* = .279; Δ*R*^2^ = .002

h: Full model *F*(4,573) = 14.31, *p* < .001; *R*^2^ = .091; Highest order unconditional interaction *F*(1, 573) = 0.10, *p* = .754; Δ*R*^2^ = .001

i: Full model *F*(4, 1378) = 34.79, *p* < .001; *R*^2^ = .092; Highest order unconditional interaction *F*(1, 1378) = 0.83, *p* = .361; Δ*R*^2^ = .001

j: Full model *F*(4, 1506) = 71.34, *p* < .001; *R*^2^ = .159; Highest order unconditional interaction *F*(1, 1506) = 18.40, *p* < .001; Δ*R*^2^ = .010

k: Full model *F*(4, 669) = 13.70, *p* < .001; *R*^2^ = .076; Highest order unconditional interaction *F*(1, 669) = 5.30, *p* = .022; Δ*R*^2^ = .007

l: Full model *F*(4, 669) = 15.14, *p* < .001; *R*^2^ = .083; Highest order unconditional interaction *F*(1, 669) = 1.74, *p* = .187; Δ*R*^2^ = .002

m: Full model *F*(4, 1115) = 32.07, *p* < .001; *R*^2^ = .103; Highest order unconditional interaction *F*(1, 1115) = 0.58, *p* =.448; Δ*R*^2^ = .001

n: Full model *F*(4, 1213) = 48.77, *p* < .001; *R*^2^ = .139; Highest order unconditional interaction *F*(1, 1213) = 37.81, *p* < .001; Δ*R*^2^ = .027

o: Full model *F*(4, 587) = 17.68, *p* < .001; *R*^2^ = .108; Highest order unconditional interaction *F*(1, 587) = 7.65, *p* = .006; Δ*R*^2^ = .012

p: Full model *F*(4, 587) = 21.46, *p* < .001; *R*^2^ = .128; Highest order unconditional interaction *F*(1, 587) = 4.13, *p* = .043; Δ*R*^2^ = .001

q: Full model *F*(4, 879) = 22.12, *p* < .001; *R*^2^ = .092; Highest order unconditional interaction *F*(1, 879) = 0.05, *p* = .818; Δ*R*^2^ = .001

r: Full model *F*(4, 954) = 62.03, *p* < .001; *R*^2^ = .206; Highest order unconditional interaction *F*(1, 954) = 1.30, *p* = .254; Δ*R*^2^ = .001

s: Full model *F*(4, 437) = 16.91, *p* < .001; *R*^2^ = .134; Highest order unconditional interaction *F*(1, 437) = 3.70, *p* = .055; Δ*R*^2^ = .007

t: Full model *F*(4, 437) = 18.08, *p* < .001; *R*^2^ = .142; Highest order unconditional interaction *F*(1, 437) = 0.62, *p* = .433; Δ*R*^2^ = .001

u: Full model *F*(4, 667) = 18.13, *p* < .001; *R*^2^ = .098; Highest order unconditional interaction *F*(1, 667) = 0.88, *p* = .349; Δ*R*^2^ = .001

v: Full model *F*(4, 704) = 32.62, *p* < .001; *R*^2^ = .156; Highest order unconditional interaction *F*(1, 704) = 18.71, *p* < .001; Δ*R*^2^ = .022

w: Full model *F*(4, 312) = 7.39, *p* < .001; *R*^2^ = .087; Highest order unconditional interaction *F*(1, 312) = 0.01, *p* = .948; Δ*R*^2^ = .001

x: Full model *F*(4, 312) = 9.84, *p* < .001; *R*^2^ = .112; Highest order unconditional interaction *F*(1, 312) = 1.34, *p* = .249; Δ*R*^2^ = .004

^*^*p* < .05; ^**^*p* < .01; ^***^*p* < .001
